# Combining ensemble models and connectivity analyses to predict wolf expected dispersal routes through a lowland corridor

**DOI:** 10.1371/journal.pone.0229261

**Published:** 2020-02-24

**Authors:** Olivia Dondina, Valerio Orioli, Elisa Torretta, Federico Merli, Luciano Bani, Alberto Meriggi

**Affiliations:** 1 Department of Earth and Environmental Sciences, University of Milano-Bicocca, Milano, Italy; 2 Department of Earth and Environmental Sciences, University of Pavia, Pavia, Italy; Institute of Geographic Sciences and Natural Resources Research Chinese Academy of Sciences, CHINA

## Abstract

The Italian wolf (*Canis lupus italicus*) population has remained isolated South of the Alps for the last few thousand years. After a strong decline, the species has recolonized the Apennines and the Western Alps, while it is currently struggling to colonize the Eastern Alps. Recently, the species was detected in a lowland park connecting the Northern Apennines to the Central Alps. If the park was able to sustain a net wolf dispersal flow, this could significantly boost the connection with the Eastern Alps and the Dinaric-Balkan population. We investigated the suitability of the park as a functional ecological corridor for the wolf through the unhospitable lowland of Northern Italy. We collected wolf occurrence data in two study areas. We modeled species distribution running a separate ensemble model for each study area and then merging the output of the models to obtain an integrated suitability map. We used this map to identify corridors for the wolf adopting a factorial least-cost path and a cumulative resistant kernel approach. The connectivity models showed that only two corridors exist in the lowland areas between the Northern Apennines and the Central Alps. The Western corridor is a blind route, while the eastern corridor passes through the park and has a continuous course. However, the models also revealed a scarce resilience of corridor connectivity in the passageways between the park and the Apennines and the Prealps, which suggests that urgent management actions are necessary to ensure the future functionality of this important corridor.

## Introduction

Large carnivores are top predators playing a key role in the maintenance of healthy and functional ecosystems [1]. Despite their ecological importance, most large carnivores suffered major declines in both population size and geographic range over the twentieth century [2]. The major causes of these declines were the loss and fragmentation of habitats, the decline of prey populations and the direct persecution by humans [3, 4, 5]. Nevertheless, in the last decades, a large-scale recovery of carnivore populations has occurred even within some of the most anthropized areas of the world, such as in Europe [6]. The recovery of large carnivores in Europe has been enhanced mainly by the institution of pan-European agreements and laws for wildlife protection [e.g. 7] and by socio-economic changes which led to an improvement in habitat quality and prey availability [8].

The wolf (*Canis lupus*) is the second most abundant large carnivore in Europe after the brown bear (*Ursus arctos*) [6]. The legal protection of the species, the conservation of natural habitats, the recovery of natural preys and its very high dispersal ability enabled the wolf to recolonize several areas of its former European range [6, 8, 9, 10, 11]. Currently, about 12,000 wolves inhabit over 800,000 km^2^ in 28 European countries [6, 12].

Despite the incredible recovery of the species, its long-term persistence in Europe is still threatened by the isolation of most populations, especially those in the Southern part of the species’ range. After the demographic decline culminated in the mid-twentieth century, the species was completely eradicated from central Europe and Scandinavia. Large wolf populations remained in the Balkans and Eastern Europe, while small and isolated populations survived in the Iberian and Italian peninsulas [12]. The genetic bottleneck triggered by the demographic decline and the geographical isolation of the Italian and Iberian wolf populations led to a dramatic decrease of their genetic diversity and to their divergence from the other European populations [13, 14, 15, 16]. A very low genetic diversity may hinder the long-term viability of a population, becoming a limiting factor when such population faces changing environmental conditions [12].

The Italian wolf population is recognized as a geographical subspecies named *Canis lupus italicus*, Altobello, 1921 [13, 17]. After population decline reached its maximum at the beginning of the ‘70s [18], wolves quickly increased in number and recolonized the whole Apennine chain moving towards the Western Italian and French Alps, where they established a new transborder population [17, 19]. In just over twenty years, the number of wolf packs in the Italian part of the Alpine population has grown from 1 to 46, most of which located in the Western Alps [1995–2018, 20]. This dramatic recovery was mainly triggered by the institution of laws for the protection of the species and by socio-economic changes which led to a general improvement in habitat quality in mountain areas, including a decrease of human density and an increase of wild ungulates [17, 18]. Overall the current Italian population includes about 1,600 wolves (about 1,300 and 300 in the Apennine and the Alpine populations, respectively) [20, 21]. While the recolonization of the Apennines and the Western Alps has been completed, the expansion of the species into the Central and Eastern Alps seems more difficult, although dispersal events towards the Central-Eastern Alps have been documented since 2010.

A stable re-colonization of the Eastern Alps would be a fundamental step for wolf conservation in Italy [22], since it would increase the genetic exchanges with the Dinaric-Balkan population. This would, in turn, generate new genotypes and increase the genetic diversity of the Italian population, with novel opportunities for local adaptations and evolution [9, 23, 24]. Since the low dispersal flow cannot guarantee a stable connection between the Eastern and the Western Alps, the presence of other connection areas between the Apennines and the Alps would be crucial. In this context, a key role could be played by the recent (2017) detection of the species in the Ticino Natural Park, after 150 years of absence from the lowland areas of Northern Italy. The Ticino Natural Park is a lowland river park longitudinally crossing the highly anthropized Northern Italian plain, which could offer a natural passage for the wolf from the Northern Apennines to the Central Alps.

In this study, we explored if the Ticino Natural Park could effectively represent a wolf dispersal route connecting the Northern Apennines to the Central Alps. Specifically, we aimed to estimate the connectivity flow both along the park and at the passages between the park and the Northern Apennines and the Central Alps. Moreover, we investigated the presence of alternative functional corridors in the area. Practically, we sought to predict areas where the wolf would have a high probability of occurrence in a wide area of Northern Italy based on an ensemble modeling approach. Then, we applied a synoptic connectivity approach [25, 26], combining distribution models with a factorial least-cost path analysis.

## Methods

### Study area

**Fig 1:**
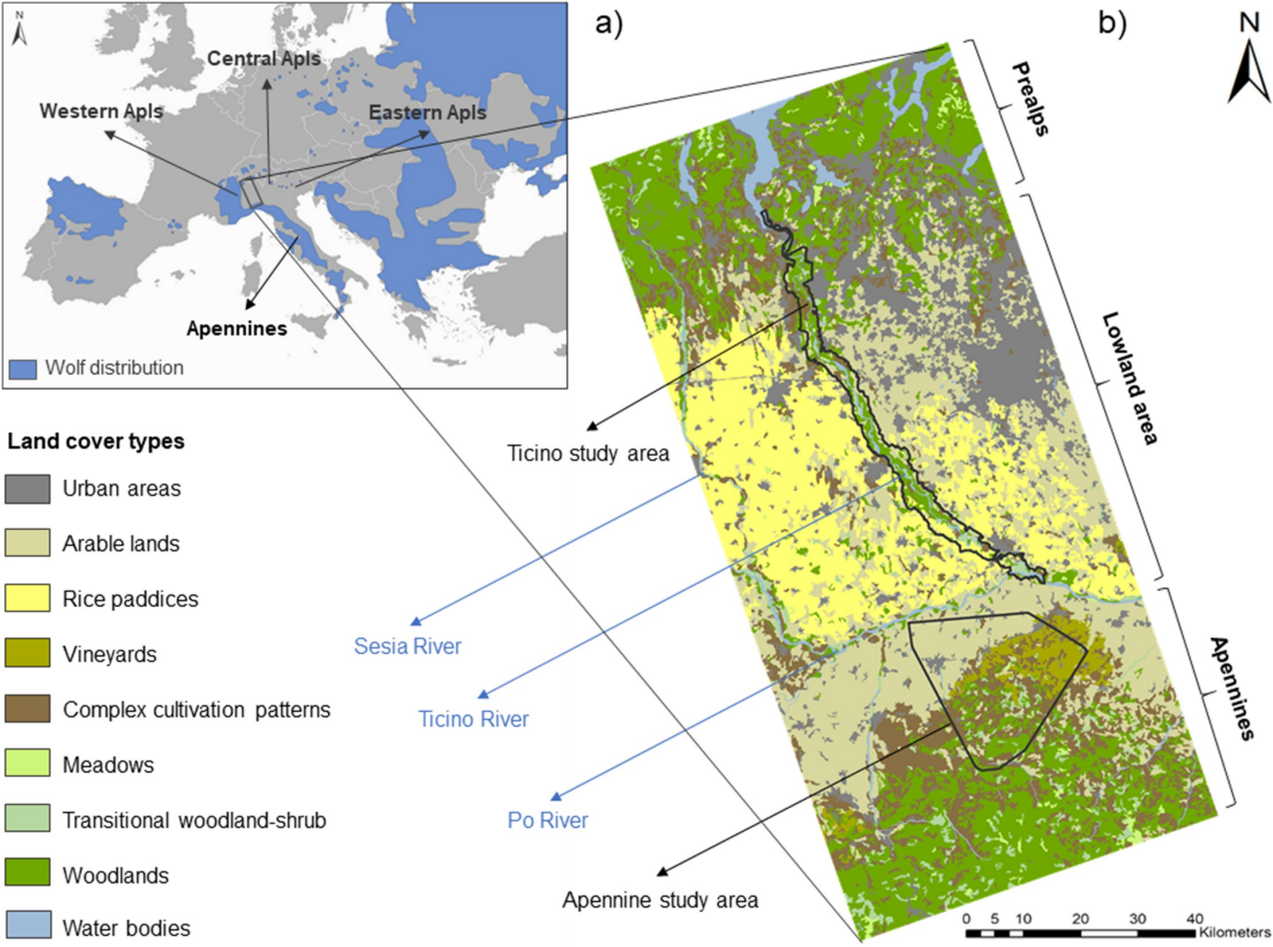
Study area. Wolf distribution in Southern Europe (a) [data from the 3rd National Report. 17 Habitat Directive, 92/43/EC uploaded with more recent results, 20] and study area in Northern Italy (b).

The study was carried out in an area of about 11,000 km^2^ in Northern Italy (centered at 45°28’ N, 8°92’ E). This wide area comprises three subregions: the Prealps in the North (about 300–2000 m a.s.l.), the lowland in the central part (below 300 m a.s.l.), and the Apennines in the South (about 300–1700 m a.s.l.) (Fig 1). The Prealps and the Apennines are characterized by a mainly continuous forest cover, while the lowland area is characterized by small and isolated residual forest fragments scattered in the intensively cultivated areas [27, 28]. The lowland is crossed from North to South by the Sesia River, by the Ticino River, and by other minor rivers, and from East to West by the Po River (Fig 1).

For this study, data were collected in two study areas: the Ticino study area within the Ticino Valley and the Apennine study area, located in the Northern portion of the Apennines (Fig 1). The Prealps were not investigated because this region has not yet been re-colonized by wolves [20].

The Ticino study area is an area of about 270 km^2^ and corresponds to the Ticino Natural Park (Lombardy and Piedmont administrative Region). It is covered for almost half of its extension by intensively cultivated crops mainly composed of rice paddies, partially flooded from April to August every year [28]. The remaining surface is largely covered by woodlands, and to a lesser extent by water bodies and built up areas. The continuous forests of the park constitute an important refuge for several mammals (57 described species), including three wild ungulate species (the wild boar *Sus scrofa*, roe deer *Capreolus capreolus* and fallow deer *Dama dama*, in a decreasing abundance order), while the wolf is the only large carnivore detected in the park.

The Northern Apennines can be divided into four different areas: the foothill area (below 300 m a.s.l.), mainly composed of intensively cultivated crops and built-up areas; the low hills (300–500 m a.s.l.), mainly composed of vineyards; the high hills (500–800 m a.s.l.), mainly covered by oak and chestnut woodlands; and the mountain area (above 800 m a.s.l.) covered by continuous beech (*Fagus sylvatica*) forests and coniferous reforestations. The Apennine study area, designed as the minimum convex polygon containing all wolf presence signs detected in the foothill, low and high hills areas, is an area of about 610 km^2^. Mountain areas were not considered because they have been occupied by stable packs since the end of the 80’s [29], while in this study we were mainly interested in gathering data from wolves colonizing new areas [30, 31]. The hilly portion of the study area hosts several mammal species, including four wild ungulate species (roe deer, wild boar, fallow deer and red deer *Cervus elaphus*, in a decreasing abundance order).

### Wolf occurrence data and land cover variables

In order to collect wolf occurrence data within the Ticino study area, 35 linear transects (mean length 8 km) covering the entire park surface were walked once per season (winter: December-February, spring: March-May, summer: June-August, autumn: September-November) in the 2017–2018 period. The transects were walked by researchers and students of the University of Milano-Bicocca and of the University of Pavia, assisted by park guards. Species presence signs (scats, tracks, predation, direct observations) were detected along the transects.

In order to collect wolf occurrence data within the Apennine study area, 18 linear transects (mean length 4.7 km) were walked once per season in the 2017–2018 period. Transect locations were defined adopting a tessellation stratified sampling design [32]. Specifically, a grid of 5 km x 5 km cells was superimposed on the study area and at least one transect was randomly identified within each cell. The transects were walked by researchers and students of the University of Pavia to detect species presence signs.

In both study areas, direct, opportunistic observations of the species during the study period were added to the presence signs systematically collected along transects.

The field work was carried out under the Law of the Republic of Italy on the Protection of Wildlife (L. 157/92). In both study areas, no specific permissions were required for walking along transects. No approval by any animal ethics committee was required because data detection did not involve sampling procedure and experimental manipulation of animals.

Nine land cover variables were selected to model wolf habitat preferences based on their relevance to the species’ ecology. The land cover variables were calculated as the fractional cover of multiple land-cover types (urban areas, arable lands, rice paddies [in the Ticino study area only], vineyards [in the Apennine study area only], complex cultivations with natural elements, meadows, transitional woodlands/shrublands, woodlands and water bodies) within a 100-m buffer around each occurrence point. The reference land use cartography was the Corine Land Cover 2018 (available at https://land.copernicus.eu/pan-european/corine-land-cover). The land cover variables were calculated on a fine spatial scale (100 m buffer) according to the results found by Ziółkowska et al. [33]. The authors demonstrated that habitat suitability models can be a useful alternative to movement data to parameterize resistance maps (which are required to perform connectivity models) provided that variables are calculated at a fine scale. Interestingly, the greater ability of single-scale fine-scale models to estimate resistance does not correspond to a best modeling performance, which is better in the case of multi-scale models. The reason for this is likely that dispersers and stable individuals behave differently with respect to habitat selection [34, 35]. When dispersers move through the landscape they largely depend on the availability and use of local resources [30], while stable individuals, which typically carry out an active habitat selection, may be also affected by broadscale patterns [36]. For the ultimate purpose of this study, the most suitable approach was therefore to consider variables calculated on a fine scale. To avoid multicollinearity among variables, we checked pairwise Pearson’s correlation coefficient between covariates and verified that no variable pair had a coefficient higher than 0.60 [1].

### Building the predictive ensemble models

We used the BIOMOD2 package [37] in R [38] to develop ensemble modeling of wolf distribution. By fitting several species distribution models, ensemble modeling decreases the uncertainty associated with using a single modeling approach and, therefore, increases the accuracy of model predictions [39]. We developed a separate ensemble model for each study area. Before running the ensemble models, a set of 300 and 500 pseudo-absence points (proportional to the study areas’ surface) were randomly generated across the Ticino and Apennine study area, respectively. To avoid the effect of sampling bias we generated the pseudo-absence points within the boundaries of the two study areas, which were exhaustively sampled. For this study, the ensemble models were implemented using three species distribution models, including generalized linear models (GLM), generalized boosted models (GBM), and a maximum entropy algorithm (MaxEnt). These species distribution models were selected based on their high predictive power [1, 40, 41, 42]. For each model, we ran three replications where 75% of the occurrence points was used as training set, while the remaining 25% was used for model evaluation. The three distribution models were evaluated for their accuracy using the area under the curve (AUC) of the receiver operating characteristic (ROC) and the true skill statistic (TSS). To integrate the output of the performed distribution models and develop the ensemble predictions, we used a weighted-averaging approach through which each single distribution model was weighted according to its predictive accuracy as independently assessed [43]. The performance of the ensemble models was evaluated using the AUC of the ROC [44, 45, 46, 47, 48]. Based on the results obtained from the models run for the two study areas, we developed two ensemble predictive maps for the whole landscape. The relative contribution of each variable in the final ensemble predictive maps for each study area was estimated by variable randomizations [43]. The predictive maps depict a gradient of suitability varying from 0 to 1000. To obtain an integrated predictive map for the whole area, the two predictive maps obtained from the ensemble models separately developed in the two study areas were superimposed and the maximum suitability value was kept for each pixel of the map. We kept the maximum suitability value for each pixel for two reasons. First, to properly appreciate the selection of sub-optimal land-cover types which can potentially be used by the wolves in areas where they are forced to use them. For instance, in the Apennine study area, woodlands (known to be the optimal habitat for the wolf, e.g. 49, 50) are concentrated in the southern part of the area, while they disappear in the North where wolves are forced to use sub-optimal land-covers, which would thus result as positively selected in this area. Conversely, in the Ticino study area woodlands have a significant cover value in the whole area not forcing wolves to use alternative land-cover types, which would thus result as not selected or avoided. This is also the reason that guided the choice of performing two separate ensemble models for the two study areas instead of a unique ensemble model, which would not have allowed us to control this effect. Second, we kept the maximum suitability value for each pixel because a part of the wolf occurrence data used as input data both for the model developed for the Ticino and Apennine study area could derive from stable and/or temporary stable individuals. In order to model species dispersal starting from these data, it would be more realistic to keep suitability values not excessively conservative [30, 33, 49]. Before integrating the two predictive maps, we set to 0 the suitability values of the pixels pertaining to land-cover types not included in the model of each study area (the vineyards in the Ticino study area and the rice paddies in the Apennine study area). Thus, the suitability values for land-cover types composing only one study area were only predicted by the model implemented in that area. Finally, the suitability value of each pixel of the final suitability map was normalized to obtain a suitability range varying from 0 to 1 [34].

### Modeling connectivity

We used the universal corridor network simulator software UNICOR [50] to model the connectivity corridors for the wolf within the landscape analyzed. The software requires a resistance map layer and a point file containing the geographic coordinates of each source location. To obtain the resistance map, we converted the integrated suitability map into a resistance map representing the permeability of the landscape to species movement using an exponential decay function [34, 51]. Because the wolf is a highly mobile species, an exponential decay function is appropriate for transforming habitat suitability into resistance values. In fact, the use of an exponential conversion means that large portions of the landscape are associated to low resistance while only highly unsuitable areas have high resistance values, leading to more flexibility in corridor location than it would be expected if the resistance map was obtained through a linear conversion of habitat suitability [1, 31]. The most suitable locations of the study area were used as source locations. Specifically, we superimposed a grid of 1 km x 1 km cells to the study area and selected only the cells composed of pixels with suitability values higher than 0.5. The centroids of these cells corresponded to the source locations for connectivity modeling. In particular, while in the Apennines and lowland area we retained all source locations, they were fictitiously rarefied in the Prealps (one centroid every 5 km) to simulate the sporadic presence of the species in this area [20]. Connectivity modeling was developed adopting a factorial least-cost path [25] and a cumulative resistant kernel approach. Factorial least-cost path analysis was used to overcome the limitation of the least-cost path approach associated with the number of sources and target points and to produce a synoptic measure of landscape connectivity [5, 26]. The resistant kernel approach shows the suitability of the whole landscape in supporting the movement of individuals [52]. Specifically, this approach calculates the least-cost dispersal Gaussian kernel around each source location up to a defined distance threshold (corresponding to the maximum dispersal distance of the species). The kernels were then combined through summation to produce a path density map [53], where the value of each pixel of the landscape corresponded to the density of the least cost paths passing through that pixel.

Because of the uncertainty about the maximum dispersal distance of the wolf, we tested two Euclidean distance thresholds: 100 km (low dispersal ability) and 850 km (high dispersal ability) [31]. We chose these two threshold distances because the mean wolf dispersal distance has been reported to range from about 13 to about 80 km [54, 55], but occasionally individuals may travel much longer distances reaching 840–866 km [55, 56]. In addition, genetic analyses performed on European wolf populations have revealed that the range of spatial influence is 650–850 km, meaning that the genetic diversity of a wolf population is influenced by populations up to 850 km apart [12].

## Results

### Predicted habitat suitability

During the study period, 96 and 71 wolf presence signs were collected within the Ticino and Apennine study area, respectively.

Among the single distribution models, GLM had the lowest performance in predicting habitat suitability in the Ticino study area, while GBM had the highest. Conversely, MaxEnt and GBM had the lowest and the highest performance, respectively, in the Apennine study area (Table 1). The output of the single distribution models for the two study areas are reported in Tables 2 and 3 (for further details see S1–S6 Figs in Supplementary material).

**Table 1:** Accuracy of species distribution models. Accuracy evaluation of the species distribution models performed in the Ticino and the Apennine study areas (AUC: area under the curve of the receiver-operating characteristic; TSS: true skill statistic).

Single models	Ticino study area		Apennine study area	
	AUC	TSS	AUC	TSS
GLM	0.682	0.308	0.774	0.458
GBM	0.814	0.465	0.874	0.611
MaxEnt	0.713	0.357	0.766	0.440

**Table 2:** Variables’ importance in the GLM, GBM and MaxEnt (Ticino study area). Score assigned to the land cover variables used to develop the GLM, GBM and MaxEnt models of wolf distribution for the Ticino study area.

Land cover variable	GLM	GBM	MaxEnt
Urban areas	0.139	0.001	0.038
Arable lands	0.000	0.066	0.061
Rice paddies	0.284	0.175	0.198
Complex cultivations with natural elements	0.000	0.069	0.095
Meadows	0.104	0.000	0.053
Transitional woodlands/shrubs	0.074	0.029	0.094
Woodlands	0.217	0.298	0.347
Water bodies	0.136	0.161	0.119

**Table 3:** Variables’ importance in the GLM, GBM and MaxEnt (Apennine study area). Score assigned to the land cover variables used to develop the GLM, GBM and MaxEnt models of wolf distribution for the Apennine study area.

Land cover variable	GLM	GBM	MaxEnt
Urban areas	0.133	0.059	0.279
Arable lands	0.254	0.278	0.873
Vineyards	0.000	0.108	0.445
Complex cultivations with natural elements	0.000	0.053	0.095
Meadows	0.000	0.000	0.309
Transitional woodlands/shrubs	0.072	0.027	0.008
Woodlands	0.374	0.256	0.008
Water bodies	0.066	0.000	0.047

As regards the ensemble model performed in the Ticino study area, the fractional cover values of rice paddies, woodlands and water bodies were the most important variables in predicting the occurrence probability of the wolf. Conversely, the most important variables in predicting the occurrence probability of the species in the Apennine area were the fractional cover values of arable lands, urban areas, woodlands and vineyards (Table 4). Both the ensemble models performed in the Ticino (AUC = 0.767) and the Apennine (AUC = 0.825) study area showed a quite good performance in predicting species occurrence.

**Table 4:** Variables’ importance in the ensemble models. Mean score assigned to the land cover variables used to develop ensemble model predictions of the wolf starting from the distribution models implemented in the Ticino and the Apennine study area.

Land cover variable	Ticino study area	Apennine study area
Urban areas	0.058	0.141
Arable lands	0.028	0.421
Rice paddies	0.309	-
Vineyards	-	0.122
Complex cultivations with natural elements	0.067	0.070
Meadows	0.049	0.001
Transitional woodlands/shrubs	0.054	0.013
Woodlands	0.155	0.131
Water bodies	0.115	0.029

**Fig 2:**
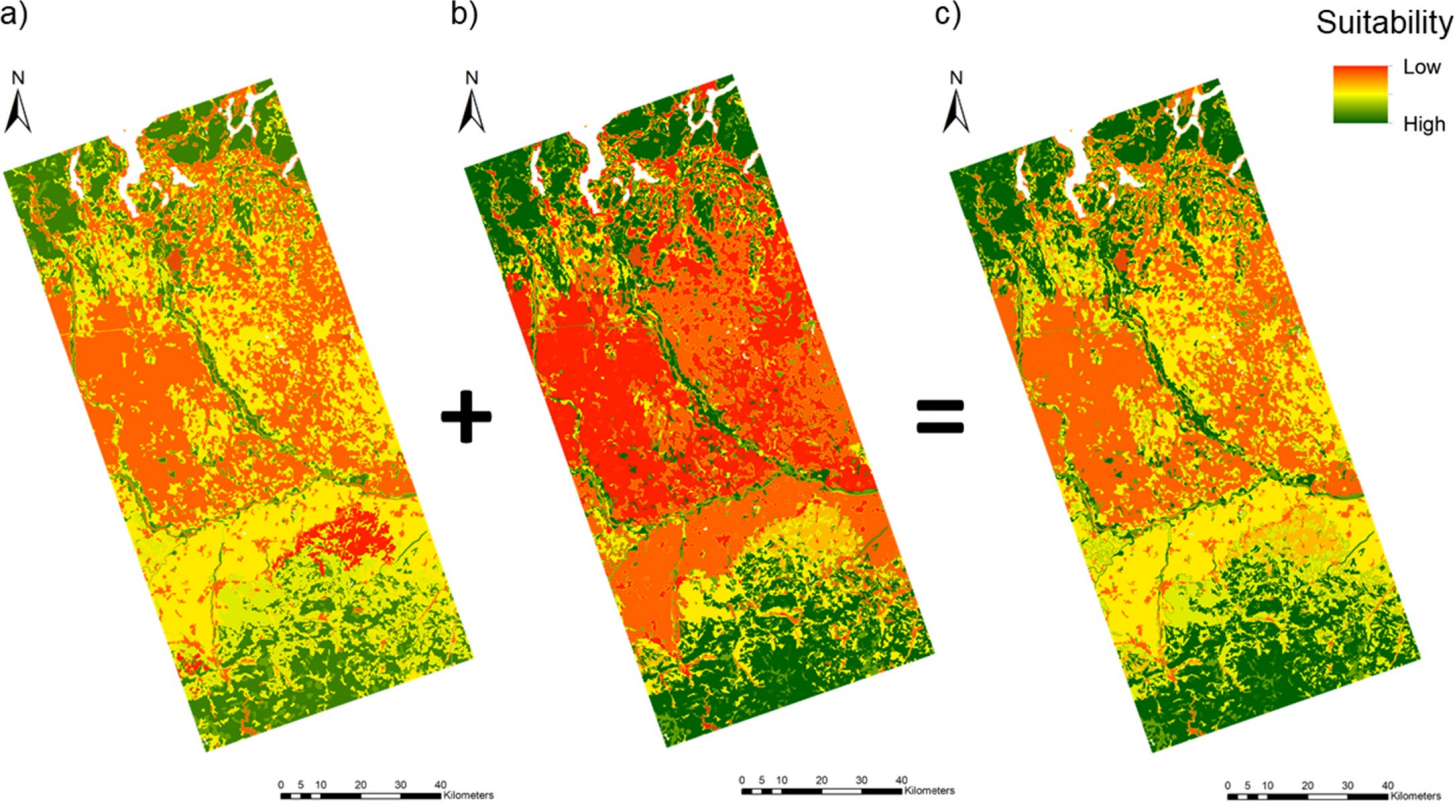
Predicted habitat suitability map. Predicted suitability (c) for the wolf based on the combined result of the ensemble models implemented in the Ticino (a) and Apennine (b) study area. White pixels: main lakes.

Predictions of the ensemble models implemented using the Ticino and Apennine data revealed that 22.8% and 32.07%, respectively, of the investigated landscape had the potential to support the presence of the wolf (considering a species occurrence probability higher than 0.5) (Fig 2A and 2B). In the integrated map, 32.3% of the landscape was suitable to support the presence of the species (Fig 2C). Specifically, the most suitable areas were located in the wooded areas of the Northern Apennines and the Prealps and along the main rivers, with a greater portion of suitable areas located along the Ticino River compared with the other rivers. Conversely, the most unsuitable areas were widely distributed in the central part of the study area and corresponded to rice paddies and urban areas (Fig 2).

### Ecological corridors

**Fig 3:**
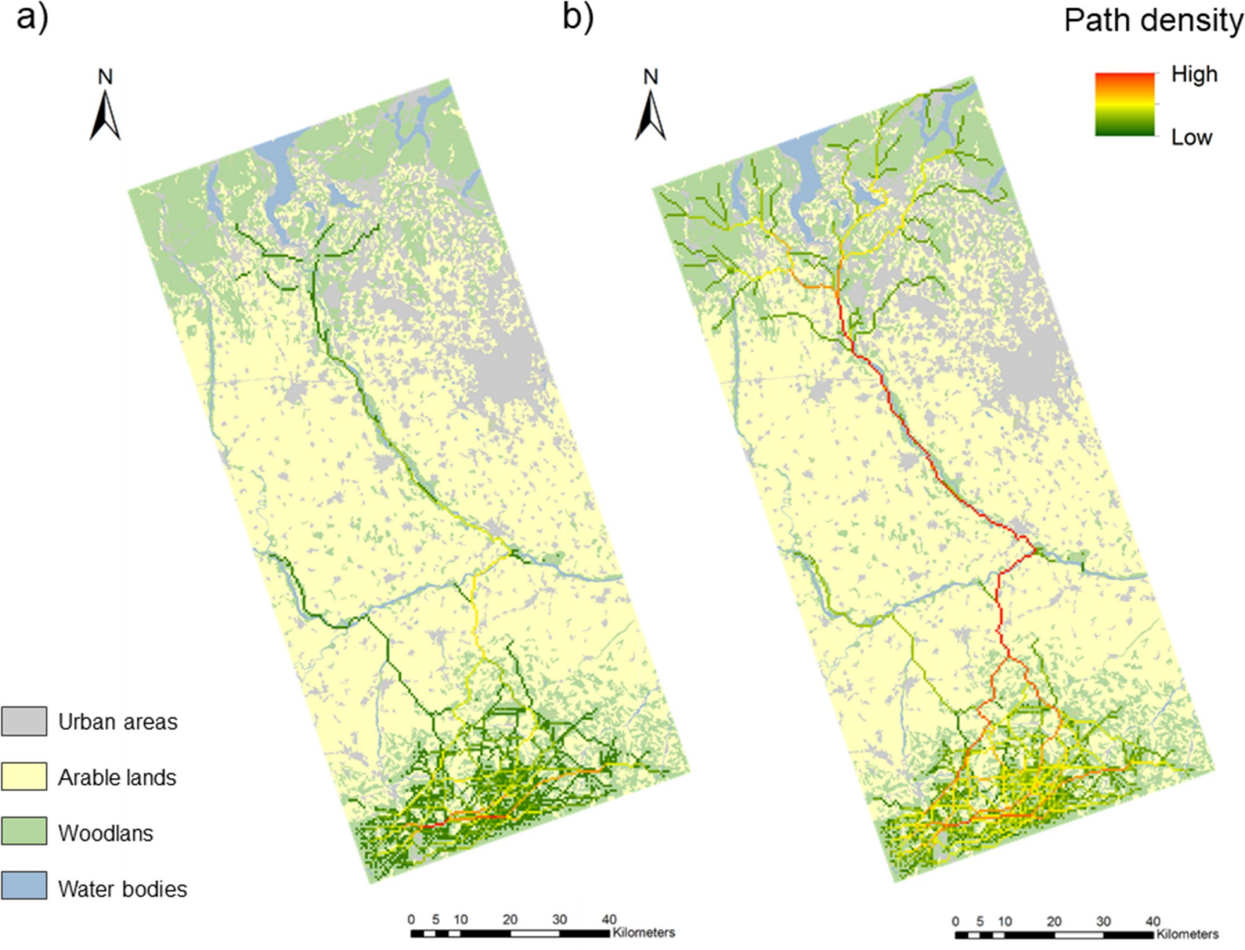
Map of corridors. Wolf corridors connecting the Apennines and the Prealps in the low (a) and high (b) dispersal ability scenarios.

The connectivity analyses were developed on 537 source locations. The predicted connectivity maps produced by the factorial least cost path models show a gradient of path density across the whole landscape (Fig 3). About 6.7% and 9.4% of the study area support wolf movements, respectively, in the low and high dispersal ability scenario, with varying degrees of probability.

The major differences between the connectivity maps representing the two dispersal scenarios concern the degree of permeability of the corridors (generally higher in the high dispersal ability scenario) rather than the number and pattern of corridors, which remains virtually unchanged except in the Northern part of the study area. However, the number and pattern of corridors in the northernmost part of the landscape, i.e. the area North to the passage between the Northern border of the Ticino Natural Park and the Prealps, are not realistic in any of the two scenarios. In fact, the Prealps are a permeable land for the wolf, but the model predicts relatively few corridors because the nodes were fictitiously rarefied in this area.

In both dispersal scenarios, the Apennine area is well connected by a very large number of corridors, characterized by a not too high path density. Leaving the hilly areas of the Apennines and moving northwards towards the plain, the models identified two corridors only. The Western corridor that heads towards the Sesia River is a blind route characterized by a low path density. Conversely, the eastern corridor that heads towards the Ticino River is characterized by a high path density and has a continuous course up to the Prealps. Along this corridor the probability of wolf passage gradually decreases moving northwards, until it abruptly drops at the passage between the Ticino Natural Park and Prealps. Once they have reached the Prealps, as reported above, wolves encounter again a wide suitable area where they can easily disperse.

## Discussion

The increase of connectivity in fragmented landscapes is essential to restore or improve the genetic exchanges between animal populations, generate new genotypes and increase genetic diversity [57, 58, 59, 60]. This is fundamental to ensure population resilience to future environmental changes. For the long-term viability of wolves in Europe, and to reach a favorable conservation status of the species (mandatory by [61]), it is crucial to preserve large populations and to enhance the dispersal flow between and within them [12]. In this study, we investigated the potential dispersal flow through the lowland between the Apennine population and the recent established Alpine population in Northern Italy, which could have important implications in increasing the genetic exchanges with the Dinaric-Balkan population and interrupting the isolation of the Italian population that has lasted for thousands of years [13, 17]. To reach this aim, we combined ensemble habitat suitability and connectivity modeling approaches [e.g. 5]. To predict the suitability degree of the whole study area, we integrated two ensemble models implemented in different study areas, in order to obtain a more realistic suitability prediction.

Based on the integration of the two predictive models, approximately one third of the study area was predicted as suitable for the wolf. However, most of the suitable areas were in the hilly and mountain areas of Northern Apennines and Prealps, were forests are widespread and continuous. Conversely, a very small portion of the central lowland area was covered by suitable areas, which were concentrated along the main rivers.

The combination of the results of the two ensemble models showed that rice paddies, arable lands and woodlands were the most important explanatory variables in predicting wolf occurrence. Rice paddies and arable lands had a strongly negative effect on species occurrence, while woodlands had a positive effect. Wolves very often use woodlands or woodland fringes to move or rest because of the shelter provided by forest cover and the high prey densities, whereas open habitats, such as rice paddies and simple arable lands, are usually avoided [55]. The wolf does not avoid agricultural areas just because of the lack of shelter and the low density of prey species, but also due to the anthropogenic disturbance typical of these habitats. Several other studies have shown that anthropogenic pressure is the variable with the most negative effect on wolf occurrence [62, 63, 64, 65]. Moreover, urban centers and agricultural areas, as well as linear infrastructures, such as roads or canals, can be important barriers for wolf movement [64]. The negative effect of such anthropogenic constructs also emerged from our analyses. In fact, the fractional cover of urban areas was another important variable with a strong negative effect on wolf occurrence. A variable with a slightly negative effect on the species was the fractional cover of vineyards. Unlike rice paddies and simple arable lands, vineyards are characterized by a more complex environmental pattern, with small woods scattered in the vineyard matrix. Finally, the relationship between wolf occurrence and the fractional cover of water bodies revealed that areas close to rivers are selected by the species. This choice depends on two fundamental requirements provided by woodland belts bordering rivers in the lowland part of the study area, i.e. protection from human disturbances and the availability of preys [62, 55, 56, 66].

The predicted connectivity maps showed that a very small portion of the study area supports wolf movements, both in the low and high dispersal ability scenarios. The corridor pattern was consistent within the maps obtained for the two dispersal ability scenarios, so we are confident that these results are highly likely. In both dispersal scenarios, most of the corridors were concentrated in Northern Apennine, but very high path densities were not detected in this area. This happened because Apennine areas are widely covered by suitable habitats for wolves [29, 62] and there are no obligatory passages in which many least cost paths converged. Moving northwards, the models identified two corridors crossing the lowland areas South to the Po River. Both corridors wind along the lowland, passing through the rare small forest patches scattered in the agricultural plain and reaching the banks of the Po River. The Western corridor moves northwards reaching the Po River and following its course for a while, before breaking off at the mouth of the Sesia River. The low probability of wolf passage throughout its whole extension makes this corridor not functional from an ecological perspective. Moreover, the abrupt change of habitat suitability between the Po River and the Sesia River, characterized by a too narrow belt of forested areas to be functional to the passage of the species, may turn this corridor into an ecological trap [67, 68]. The Eastern corridor has a continuous course up to the Prealps and is characterized by a high path density both in the crossing area of the Southern agricultural plain and along the entire course of the Ticino River, while path density abruptly decreases at the crossing point between the Ticino River and the Prealps. On the one hand, the continuity and high permeability of this corridor encourage optimism regarding the existence of a functional ecological connection between the Northern Apennine and the Central Alps, passing through the Ticino Natural Park. On the other hand, the results predicted by the factorial least cost path models showed at least two critical points. First of all, the passage between the Northern Apennines and the Southern border of the Ticino Natural Park is supported by one corridor only, and this can be problematic from a conservation point of view because dispersers seldom follow a single optimal route in a landscape [69]. Moreover, if for any reason the only corridor present in a landscape was no longer functional to the passage of the species, the connectivity of the area would drastically collapse. Redundant corridors are fundamental to strengthen the effectiveness and resilience of the landscape ecological network [70] and to the long-term maintenance of connectivity even in the face of future environmental changes [71]. The second critical point regards the difficult passage from the Northern border of the Ticino Natural Park to the Prealps, which could compromise the functionality of the whole corridor. Here, the permeability of the corridor drops abruptly because of the large number of built-up areas and roads crossing the area [66, 72, 73]. This hypothesis is supported by the finding of a wolf hit by a car exactly at this crossing point in 2012.

From a management point of view, in order to ensure the long-term maintenance of the effectiveness of the Ticino Natural Park as a functional corridor for the wolf it is necessary to enhance connectivity both at the passage between the Northern Apennines and the Southern border of the park and, in particular, between the northern border of the park and the Prealps. Specifically, at the passage between the Northern Apennines and the Southern border of the park, connectivity should be enhanced by reducing the resistance of the agricultural matrix, for example by setting aside fields, planting hedgerows or restoring the banks of the numerous small streams crossing the areas, which already represent natural underpasses for crossing motorways. As regards the Northern passage between the Park and the Prealps, we did not have data on the location of viaducts or drainage channels under traffic roads. Such data should be considered in a local scale study aimed at evaluating in detail the permeability conditions of this crossing area and, eventually, at identifying priority areas to defragment barriers through overpasses and underpasses [55].

## Conclusions

In conclusion, the Ticino Natural Park seems to be a functional corridor for sustaining the wolf dispersal flow from the Northern Apennines to the Central Alps, which would increase the probability of a future stable genetic exchange between the Italian and the Dinaric-Balkan populations. However, the analyses also showed that this corridor is fragile and not resilient to possible environmental changes. The Ticino Natural Park is probably the only functional corridor that connects the Apennines to the Central or Eastern Alps by crossing the wide anthropized agricultural plain of Northern Italy. All the other longitudinal rivers crossing this area, even if included in protected areas, are often bordered by a very narrow and fragmented strip of natural vegetation. If, for any reason, the Ticino Natural Park was no longer functional to sustain the wolf dispersal flow, the connectivity across the whole lowland area of Northern Italy would drastically collapse. Therefore, it is crucial to maintain the functionality of the corridor, not only along the Ticino River but also, and above all, in the passageways between the park and the source (Apennines) and destination (Prealps) areas.

## Supporting information

S1 File Data. Presence and pseudo-absence wolf locations in the Apennine and Ticino study area.
(XLSX)

S1 Fig GLM plots (Ticino study area). Plots representing the relationship between the wolf occurrence probability (y axes) and the fractional cover of the land cover variables (x axes) obtained from the GLM run for the Ticino study area.
(TIF)

S2 Fig GBM plots (Ticino study area). Plots representing the relationship between the wolf occurrence probability (y axes) and the fractional cover of the land cover variables (x axes) obtained from the GBM run for the Ticino study area.
(TIF)

S3 Fig MaxEnt plots (Ticino study area). Plots representing the relationship between the wolf occurrence probability (y axes) and the fractional cover of the land cover variables (x axes) obtained from the MaxEnt model run for the Ticino study area.
(TIF)

S4 Fig GLM plots (Apennine study area). Plots representing the relationship between the wolf occurrence probability (y axes) and the fractional cover of the land cover variables (x axes) obtained from the GLM run for the Apennine study area.
(TIF)

S5 Fig GBM plots (Apennine study area). Plots representing the relationship between the wolf occurrence probability (y axes) and the fractional cover of the land cover variables (x axes) obtained from the GBM run for the Apennine study area.
(TIF)

S6 Fig MaxEnt plots (Apennine study area). Plots representing the relationship between the wolf occurrence probability (y axes) and the fractional cover of the land cover variables (x axes) obtained from the MaxEnt model run for the Apennine study area.
(TIF)

## References

[1] Khosravi R, Hemami MR, Cushman SA. Multispecies assessment of core areas and connectivity of desert carnivores in central Iran. Divers Distrib. 2018; 24: 193–207.

[2] Ripple WJ, Estes JA, Beschta RL, Wilmers CC, Ritchie EG, Hebblewhite M, et al Status and ecological effects of the world’s largest carnivores. Science. 2014; 343: 1241484 10.1126/science.1241484 24408439

[3] Breitenmoser U. Large predators in the Alps: the fall and rise of Man’s competitors. Biol Conserv. 1998; 83: 279–289.

[4] Kabir M, Hameed S, Ali H, Bosso L, Din JU, Bischof R, et al Habitat suitability and movement corridors of grey wolf (*Canis lupus*) in Northern Pakistan. PloS one. 2017; 12: e0187027 10.1371/journal.pone.0187027 29121089 PMC5679527

[5] Shahnaseri G, Hemami MR, Khosravi R, Malakoutikhah S, Omidi M, Cushman S. Contrasting use of habitat, landscape elements, and corridors by grey wolf and golden jackal in central Iran. Landsc Ecol. 2019; 34: 1263–1277.

[6] Chapron G, Kaczensky P, Linnell JD, von Arx M, Huber D, Andrén H, et al Recovery of large carnivores in Europe’s modern human-dominated landscapes. Science. 2014; 346: 1517–1519. 10.1126/science.1257553 25525247

[7] Convention on the Conservation of European Wildlife and Natural Heritage. 1979. http://conventions.coe.int/Treaty/en/Treaties/html/104.htm.

[8] Linnell JDC, Zachos FE. Status and distribution patterns of European ungulates: genetics, population history and conservation In: Putman R, Apollonio M, Andersen R, editors. Ungulate Management in Europe: Problems and Practices. Cambridge: Cambridge University Press; 2011 pp. 12–53.

[9] Randi E. Genetics and conservation of wolves *Canis lupus* in Europe. Mammal Rev. 2011; 41: 99–111.

[10] Leonard JA. Ecology drives evolution in grey wolves. Evol Ecol Res. 2014; 16: 461–473.

[11] Gilroy JJ, Ordiz A, Bischof R. Carnivore coexistence: value the wilderness. Science. 2015; 347: 382. 10.1126/science.347.6220.382-a 25613880

[12] Hindrikson M, Remm J, Pilot M, Godinho R, Stronen AV, Baltrūnaité L, et al Wolf population genetics in Europe: a systematic review, meta‐analysis and suggestions for conservation and management. Biol Rev. 2017; 92: 1601–1629. 10.1111/brv.12298 27682639

[13] Lucchini V, Galov A, Randi E. Evidence of genetic distinction and long-term population decline in wolves (*Canis lupus*) in the Italian Apennines. Mol Ecol. 2004; 13: 523–536. 10.1046/j.1365-294x.2004.02077.x 14871358

[14] Sastre N, Vilà C, Salinas M, Bologov VV, Urios V, Sánchez A, et al Signatures of demographic bottlenecks in European wolf populations. Conserv Genet. 2011; 12: 701–712.

[15] Stronen AV, Jędrzejewska B, Pertoldi C, Demontis D, Randi E, Niedziałkowska M, et al North-south differentiation and a region of high diversity in European wolves (*Canis lupus*). PLoS One. 2013; 8: e76454 10.1371/journal.pone.0076454 24146871 PMC3795770

[16] Pilot M, Greco C, vonHoldt BM, Jędrzejewska B, Randi E, Jędrzejewski W, et al Genome-wide signatures of population bottlenecks and diversifying selection in European wolves. Heredity. 2014; 112: 428–442. 10.1038/hdy.2013.122 24346500 PMC3966127

[17] Fabbri E, Miquel C, Lucchini V, Santini A, Caniglia R, Duchamp C, et al From the Apennines to the Alps: colonization genetics of the naturally expanding Italian wolf (*Canis lupus*) population. Mol Ecol. 2007; 16: 1661–1671. 10.1111/j.1365-294X.2007.03262.x 17402981

[18] Boitani L. Wolf conservation and recovery In: Mech LD, Boitani L, editors. Wolves: Behavior, Ecology and Conservation. Chicago: University of Chicago Press; 2003 pp. 317–340.

[19] Marucco F, Avanzinelli E, Boitani L. Non-invasive integrated sampling design to monitor the wolf population in Piemonte, Italian Alps. Hystrix. 2012; 23: 5–13.

[20] Marucco F, Avanzinelli E, Bassano B, Bionda R, Bisi F, Calderola S, et al. La popolazione di lupo sulle Alpi Italiane 2014–2018. Technical report, LIFE 12 NAT/IT/00080 WOLFALPS–Actions A4 and D1. 2018.

[21] Galaverni M, Caniglia R, Fabbri E, Milanesi P, Randi E. One, no one, or one hundred thousand: how many wolves are there currently in Italy? Mamm Res. 2016; 61: 13–24.

[22] Genovesi P. Piano d'azione nazionale per la conservazione del Lupo (*Canis lupus*). 2002.

[23] Hedrick PW, Fredrickson R. Genetic rescue guidelines with examples from Mexican wolves and Florida panthers. Conserv Genet. 2010; 11: 615–626.

[24] Fabbri E, Caniglia R, Kusak J, Galov A, Gomerčić T, Arbanasić H, et al Genetic structure of expanding wolf (*Canis lupus*) populations in Italy and Croatia, and the early steps of the recolonization of the Eastern Alps. Mamm Biol. 2014; 79: 138–148.

[25] Cushman SA, McKelvey KS, Schwartz MK. Use of empirically derived source-destination models to map regional conservation corridors. Conserv Biol. 2009; 23: 368–376. 10.1111/j.1523-1739.2008.01111.x 19016821

[26] Cushman SA, Lewis JS, Landguth EL. Why did the bear cross the road? Comparing the performance of multiple resistance surfaces and connectivity modeling methods. Diversity. 2014; 6: 844–854.

[27] Chiatante G, Dondina O, Lucchelli M, Bani L, Meriggi A. Habitat selection of European badger *Meles meles* in a highly fragmented forest landscape in northern Italy: the importance of hedgerows and agro-forestry systems. Hystrix. 2017; 28: 247–252.

[28] Dondina O, Orioli V, D'Occhio P, Luppi M, Bani L. How does forest species specialization affect the application of the island biogeography theory in fragmented landscapes? J Biogeogr. 2017; 44: 1041–1052.

[29] Meriggi A, Rosa P, Brangi A, Matteucci C. Habitat use and diet of the wolf in Northern Italy. Acta Theriol. 1991; 36:141–151.

[30] Zeller KA, McGarigal K, Whiteley AR. Estimating landscape resistance to movement: a review. Landsc Ecol. 2012; 27: 777–797.

[31] Wan HY, Cushman SA, Ganey JL. Improving habitat and connectivity model predictions with multi-scale resource selection functions from two geographic areas. Landsc Ecol. 2019; 34: 503–519.

[32] Barabesi L, Franceschi S. Sampling properties of spatial total estimators under tessellation stratified designs. Environmetrics. 2011; 22: 271–278.

[33] Ziółkowska E, Ostapowicz K, Radeloff VC, Kuemmerle T, Sergiel A, Zwijacz-Kozica T, et al Assessing differences in connectivity based on habitat versus movement models for brown bears in the Carpathians. Landsc Ecol. 2016; 31: 1863–1882.

[34] Mateo Sánchez MC, Balkenhol N, Cushman SA, Perez T, Dominguez A, Saura S. A comparative framework to infer landscape effects on population genetic structure: are habitat suitability models effective in explaining gene flow? Landsc Ecol. 2015a; 30: 1405–1420.

[35] Dondina O, Orioli V, Colli L, Luppi M, Bani L. Ecological network design from occurrence data by simulating species perception of the landscape. Landsc Ecol. 2018a; 33: 275–287.

[36] Mateo Sánchez MC, Cushman SA, Saura S. Scale dependence in habitat selection: the case of the endangered brown bear (*Ursus arctos*) in the Cantabrian Range (NW Spain). Int J Geogr Inf Sci. 2013; 28: 1531–1546.

[37] Thuiller W, Georges D, Engler R, Breiner F. biomod2: Ensemble platform for species distribution modeling. R package version 3.3–7. 2016.

[38] R Core Team. R: A language and environment for statistical computing. R Foundation for Statistical Computing, Vienna, Austria 2017 URL https://www.R-project.org/.

[39] Araújo MB, New M. Ensemble forecasting of species distributions. Trends Ecol Evol. 2007; 22: 42–47. 10.1016/j.tree.2006.09.010 17011070

[40] Bosso L, Ancillotto L, Smeraldo S, D’Arco S, Migliozzi A, Conti P, et al Loss of potential bat habitat following a severe wildfire: a model-based rapid assessment. Int J Wildland Fire. 2018; 27: 756–769.

[41] Fois M, Bacchetta G, Cuena-Lombrana A, Cogoni D, Pinna MS, Sulis E, et al Using extinctions in species distribution models to evaluate and predict threats: a contribution to plant conservation planning on the island of Sardinia. Environ Conserv. 2018; 45: 11–19.

[42] Maiorano L, Chiaverini L, Falco M, Ciucci P. Combining multi-state species distribution models, mortality estimates, and landscape connectivity to model potential species distribution for endangered species in human dominated landscapes. Biol Conserv. 2019; 237: 19–27.

[43] Thuiller W, Lafourcade B, Engler R, Araújo MB. BIOMOD–a platform for ensemble forecasting of species distributions. Ecography. 2009; 32: 369–373.

[44] Marmion M, Parviainen M, Luoto M, Heikkinen RK, Thuiller W. Evaluation of consensus methods in predictive species distribution modelling. Divers Distrib. 2009; 15: 59–69.

[45] Rodríguez‐Soto C, Monroy‐Vilchis O, Maiorano L, Boitani L, Faller JC, Briones MÁ, et al Predicting potential distribution of the jaguar (Panthera onca) in Mexico: identification of priority areas for conservation. Divers Distrib. 2011; 17: 350–361.

[46] Fernandes RF, Honrado JP, Guisan A, Roxo A, Alves P, Martins J, et al Species distribution models support the need of international cooperation towards successful management of plant invasions. J Nat Conserv. 2019; 49: 85–94.

[47] Mohammadi S, Ebrahimi E, Moghadam MS, Bosso L. Modelling current and future potential distributions of two desert jerboas under climate change in Iran. Ecol Inform. 2019; 52: 7–13.

[48] Scherrer D, Christe P, Guisan A. Modelling bat distributions and diversity in a mountain landscape using focal predictors in ensemble of small models. Divers Distrib. 2019; 25: 770–782.

[49] Abrahms B, Sawyer SC, Jordan NR, McNutt JW, Wilson AM, Brashares JS. Does wildlife resource selection accurately inform corridor conservation?. J Appl Ecol. 2017; 54: 412–422.

[50] Landguth E, Hand B, Glassy J, Cushman S, Sawaya M. UNICOR: a species connectivity and corridor network simulator. Ecography. 2012; 35: 9–14.

[51] Mateo Sánchez MC, Balkenhol N, Cushman SA, Pérezm T, Domínguez A, Saura S. Estimating effective landscape distances and movement corridors: comparison of habitat and genetic data. Ecosphere. 2015b; 6:1–16.

[52] Cushman SA, Lewis JS, Landguth EL. Evaluating the intersection of a regional wildlife connectivity network with highways. Mov Ecol. 2013a; 1: 12 10.1186/2051-3933-1-12 25709825 PMC4337767

[53] Cushman SA, Landguth EL, Flather CH. Evaluating population connectivity for species of conservation concern in the American Great Plains. Biodivers Conserv. 2013b; 22: 2583–2605.

[54] Boitani L. Wolf management in intensively used areas of Italy In: Harrington FH, Paquet PC, editors. Wolves of the word. Perspectives of behavior, ecology and conservation. Park Ridge (NJ): Noyes Publications; 1982 pp. 158–172.

[55] Rodríguez-Freire M, Crecente-Maseda R. Directional connectivity of wolf (*Canis lupus*) populations in northwest Spain and anthropogenic effects on dispersal patterns. Environ Model Assess. 2008; 13: 35.

[56] Boitani L. Action plan for the conservation of the wolves (*Canis lupus*) in Europe Nature and environment. 118 Council of Europe Publishing; 2000.

[57] Lowe WH, Allendorf FW. What can genetics tell us about population connectivity? Mol Ecol. 2010; 19: 3038–3051. 10.1111/j.1365-294X.2010.04688.x 20618697

[58] Bani L, Pisa G, Luppi M, Spilotros G, Fabbri E, Randi E, et al Ecological connectivity assessment in a strongly structured fire salamander (Salamandra salamandra) population. Ecol Evol. 2015; 5: 3472–3485. 10.1002/ece3.1617 26380679 PMC4569041

[59] Bani L, Orioli V, Pisa G, Fagiani S, Dondina O, Fabbri E, et al Population genetic structure and sex-biased dispersal of the hazel dormouse (Muscardinus avellanarius) in a continuous and in a fragmented landscape in central Italy. Conserv Genet. 2017; 18: 261–274.

[60] Bani L, Orioli V, Pisa G, Dondina O, Fagiani S, Fabbri E, et al Landscape determinants of genetic differentiation, inbreeding and genetic drift in the hazel dormouse (Muscardinus avellanarius). Conserv Genet. 2018; 19: 283–296.

[61] Council Directive 92/43/EEC of 21 May 1992 on the conservation of natural habitats and of wild fauna and flora. 1992. http://eur-lex.europa.eu/legal-content/EN/TXT/?uri=CELEX:31992L0043.

[62] Massolo A, Meriggi A. Factors affecting habitat occupancy by wolves in northern Apennines (northern Italy): a model of habitat suitability. Ecography. 1998; 21: 97–107.

[63] Eggermann J, da Costa GF, Guerra AM, Kirchner WH, Petrucci-Fonseca F. Presence of Iberian wolf (*Canis lupus* signatus) in relation to land cover, livestock and human influence in Portugal. Mamm Biol. 2011; 76: 217–221.

[64] Jędrzejewski W, Niedzialkowska M, Nowak S, Jędrzejewska B. Habitat variables associated with wolf (Canis lupus) distribution and abundance in northern Poland. Divers Distrib. 2004; 10: 225–233.

[65] Imbert C, Caniglia R, Fabbri E, Milanesi P, Randi E, Serafini M, et al Why do wolves eat livestock?: Factors influencing wolf diet in northern Italy. Biol Conserv. 2016; 195: 156–168.

[66] Cayuela L. Habitat evaluation for the Iberian wolf Canis lupus in Picos de Europa National Park, Spain. Appl Geogr. 2004; 24: 199–215.

[67] Delibes M, Gaona P, Ferreras P. Effects of an attractive sink leading into maladaptive habitat selection. Am Nat. 2001; 158: 277–285. 10.1086/321319 18707324

[68] Schlaepfer MA, Runge MC, Sherman PW. Ecological and evolutionary traps. Trends Ecol Evol. 2002; 17: 474–480.

[69] Pinto N, Keitt TH. Beyond the least-cost path: evaluating corridor redundancy using a graph-theoretic approach. Landsc Ecol. 2009; 24:253–266.

[70] Dondina O, Saura S, Bani L, Mateo-Sánchez MC. Enhancing connectivity in agroecosystems: focus on the best existing corridors or on new pathways? Landsc Ecol. 2018b; 33: 1741–1756.

[71] McRae BH, Dickson BG, Keitt TH, Shah VB. Using circuit theory to model connectivity in ecology, evolution, and conservation. Ecology. 2008; 89: 2712–2724. 10.1890/07-1861.1 18959309

[72] Jędrzejewski W, Niedziałkowska M, Myslajek RW, Nowak S, Jędrzejewska B. Habitat selection by wolves Canis lupus in the uplands and mountains of southern Poland. Acta Theriol. 2005; 50: 417–428.

[73] Kaartinen S, Kojola I, Colpaert A. Finnish wolves avoid roads and settlements. Ann Zool Fennici. 2005; 42: 523–532.

