# Supplementary figures and images for: Combining ensemble models and connectivity analyses to predict wolf expected dispersal routes through a lowland corridor
Source: PLoS One. 2020 Feb 24;15(2):e0229261. doi: 10.1371/journal.pone.0229261 (PMC7039448; doi:10.1371/journal.pone.0229261)

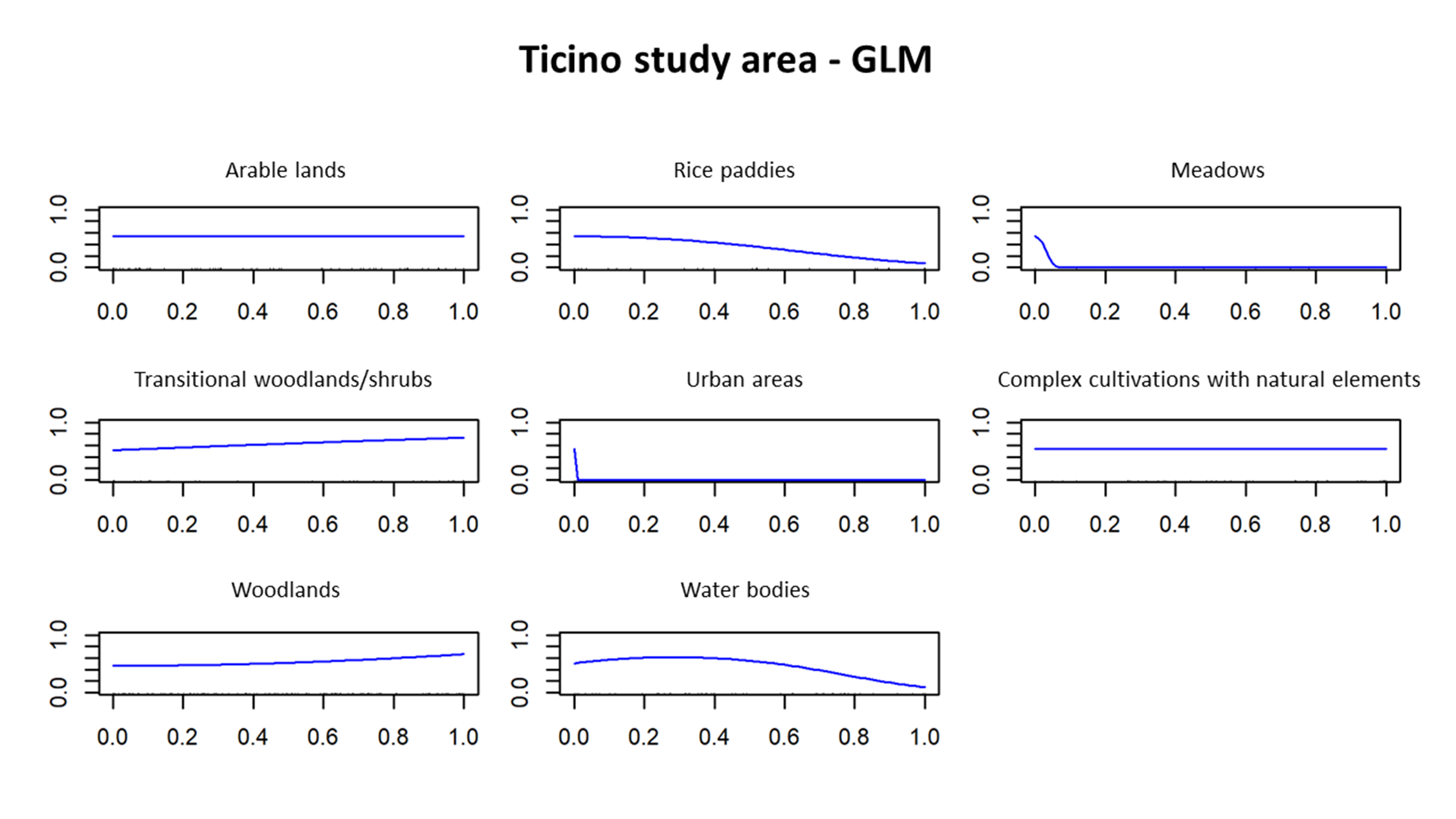

Supplement: S1 Fig — Plots representing the relationship between the wolf occurrence probability (y axes) and the fractional cover of the land cover variables (x axes) obtained from the GLM run for the Ticino study area. (TIF) [file pone.0229261.s002.tif]

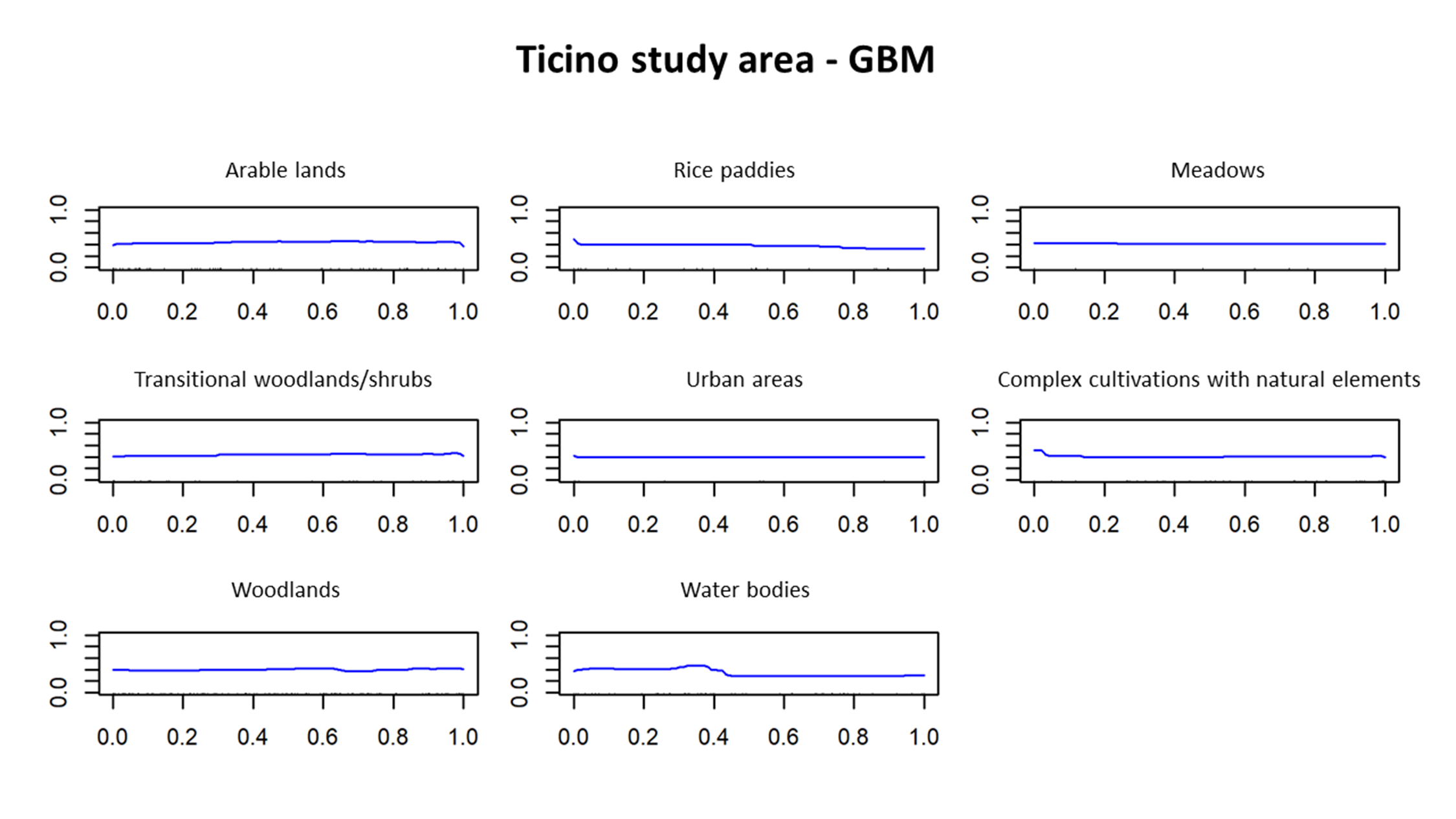

Supplement: S2 Fig — Plots representing the relationship between the wolf occurrence probability (y axes) and the fractional cover of the land cover variables (x axes) obtained from the GBM run for the Ticino study area. (TIF) [file pone.0229261.s003.tif]

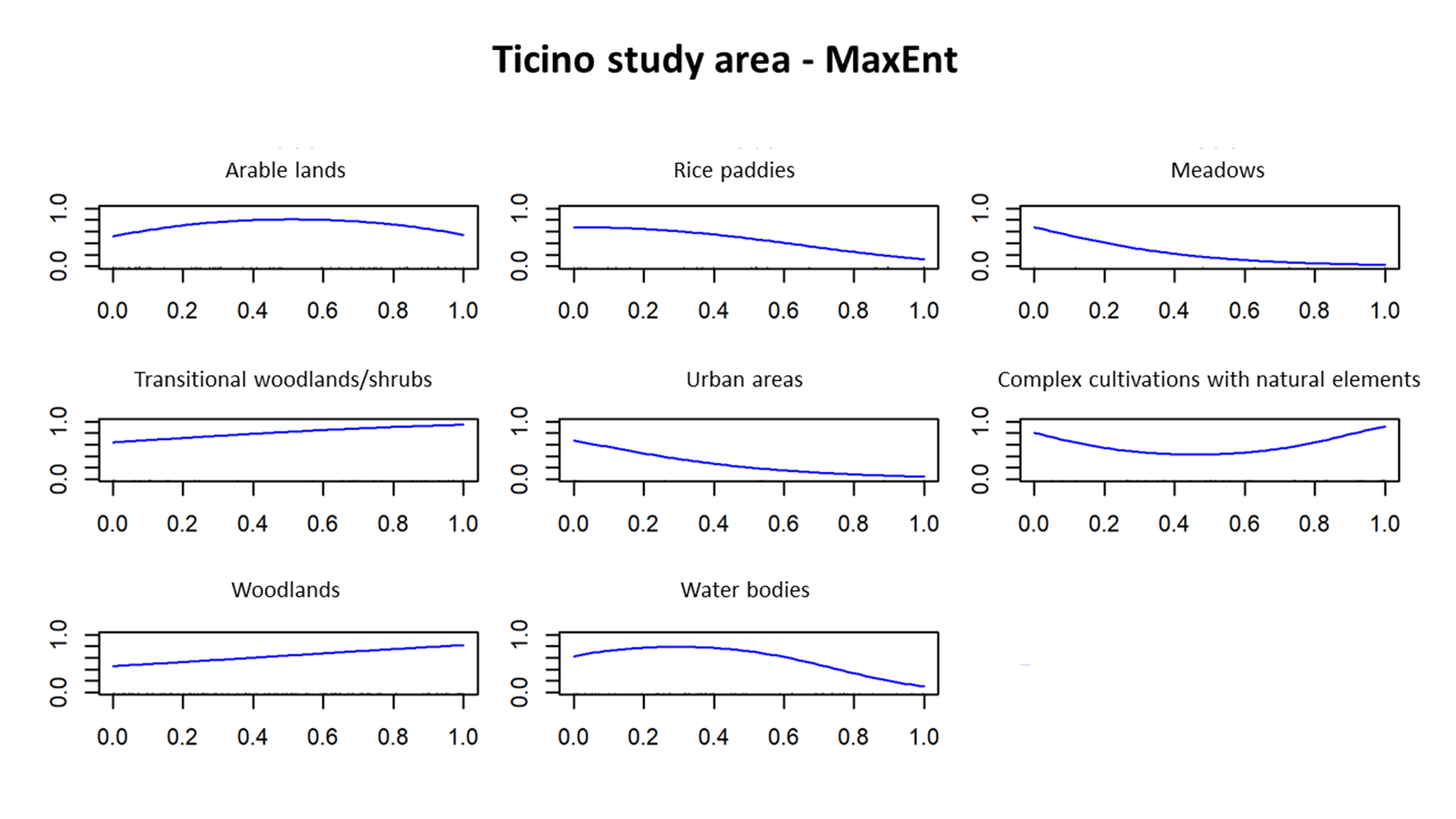

Supplement: S3 Fig — Plots representing the relationship between the wolf occurrence probability (y axes) and the fractional cover of the land cover variables (x axes) obtained from the MaxEnt model run for the Ticino study area. (TIF) [file pone.0229261.s004.tif]

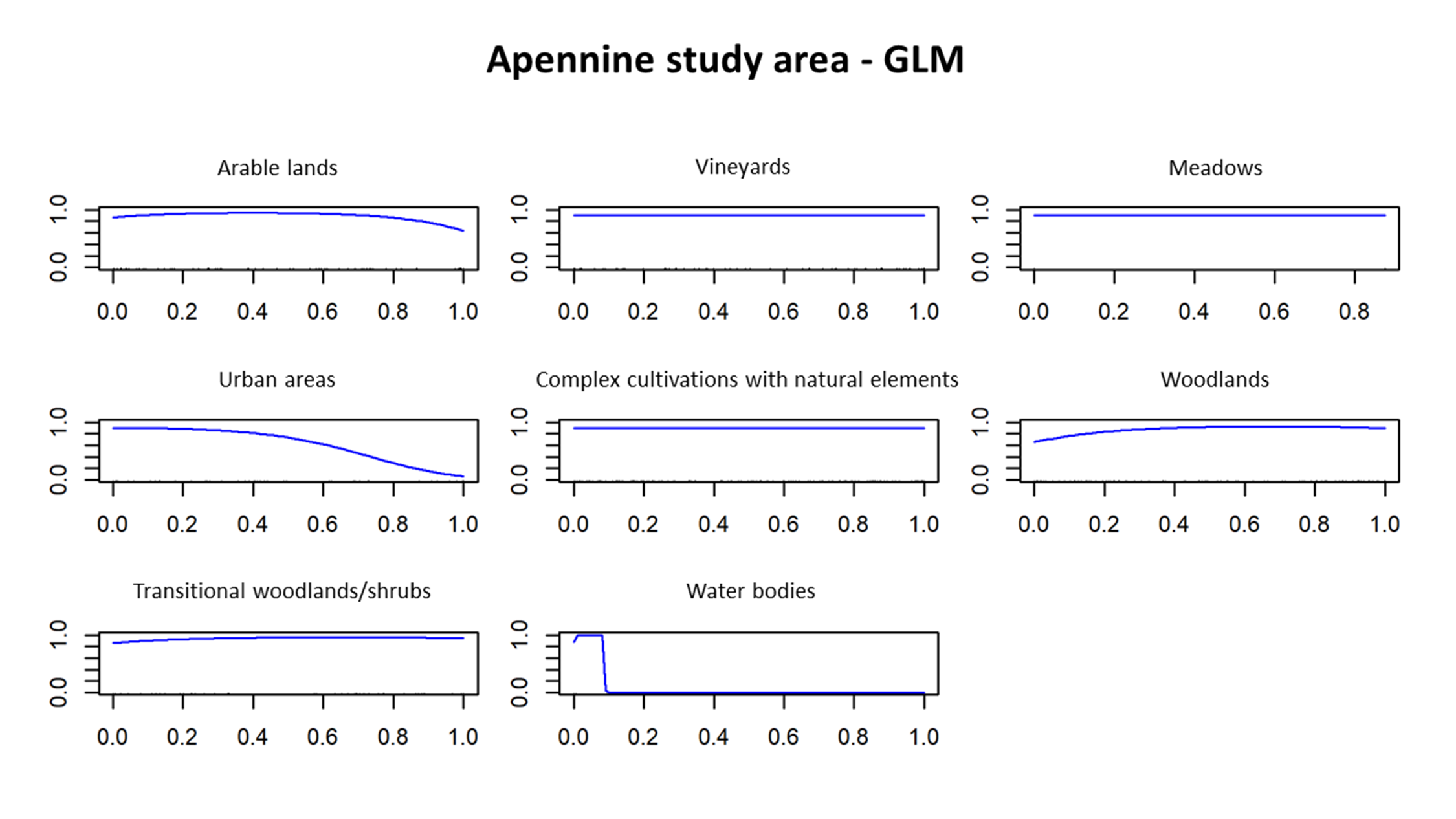

Supplement: S4 Fig — Plots representing the relationship between the wolf occurrence probability (y axes) and the fractional cover of the land cover variables (x axes) obtained from the GLM run for the Apennine study area. (TIF) [file pone.0229261.s005.tif]

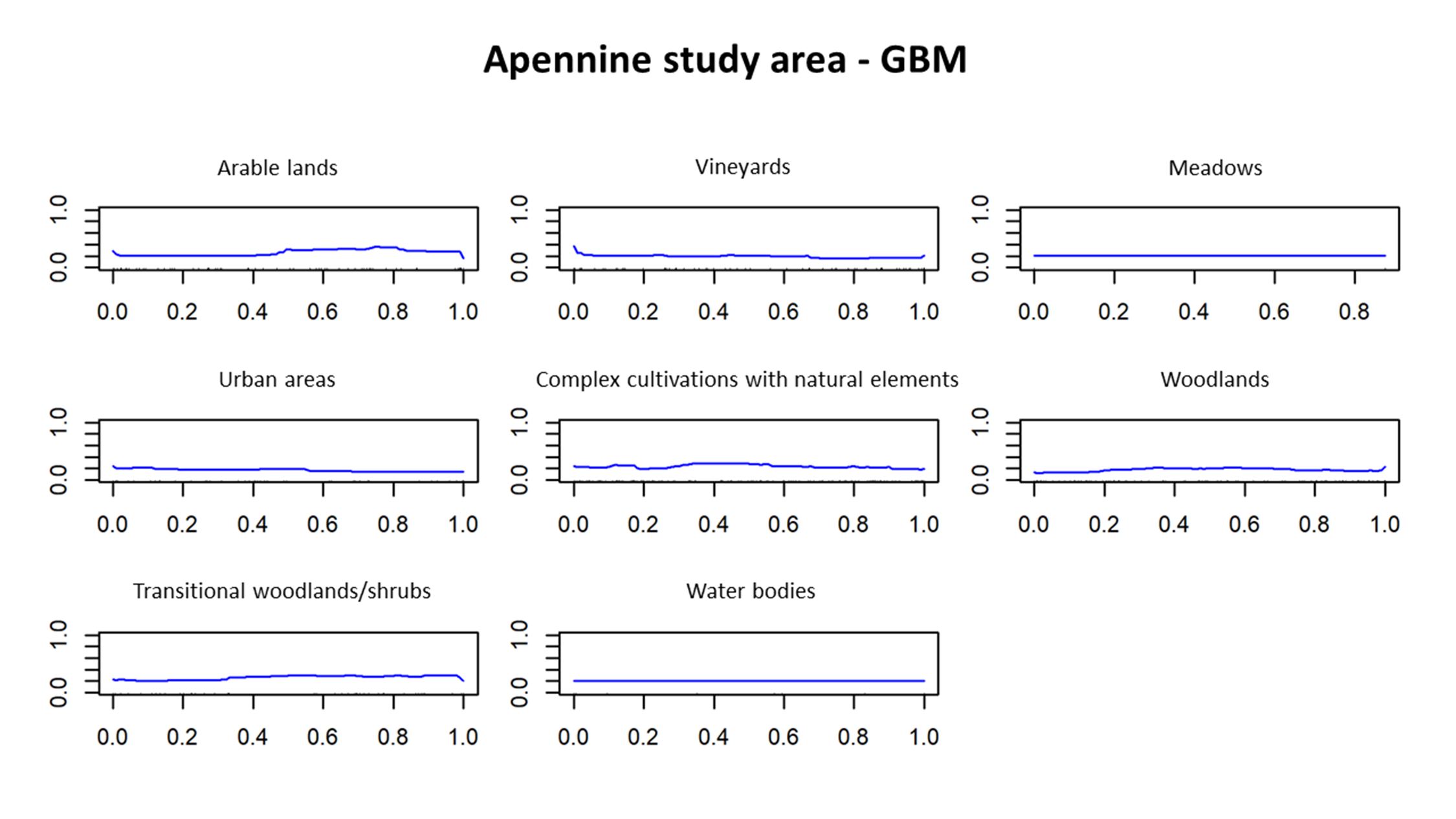

Supplement: S5 Fig — Plots representing the relationship between the wolf occurrence probability (y axes) and the fractional cover of the land cover variables (x axes) obtained from the GBM run for the Apennine study area. (TIF) [file pone.0229261.s006.tif]

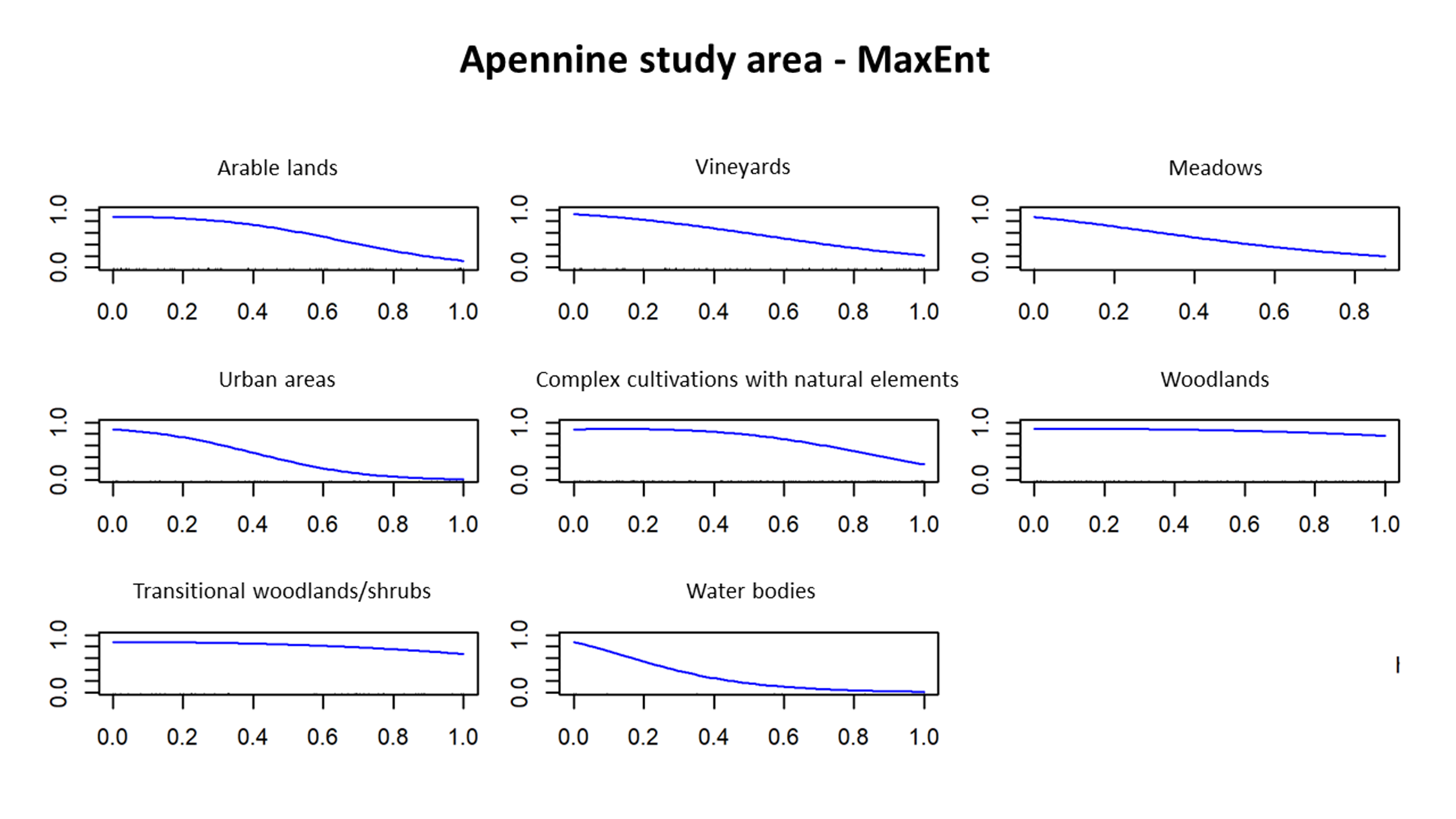

Supplement: S6 Fig — Plots representing the relationship between the wolf occurrence probability (y axes) and the fractional cover of the land cover variables (x axes) obtained from the MaxEnt model run for the Apennine study area. (TIF) [file pone.0229261.s007.tif]
